# Beyond Stress Ischemia: Unveiling the Multifaceted Nature of Coronary Vulnerable Plaques Using Cardiac Computed Tomography

**DOI:** 10.3390/jcm13144277

**Published:** 2024-07-22

**Authors:** Gianluigi Napoli, Saima Mushtaq, Paolo Basile, Maria Cristina Carella, Daniele De Feo, Michele Davide Latorre, Andrea Baggiano, Marco Matteo Ciccone, Gianluca Pontone, Andrea Igoren Guaricci

**Affiliations:** 1 University Cardiologic Unit, Interdisciplinary Department of Medicine, University of Bari “Aldo Moro”, Polyclinic University Hospital, 70124 Bari, Italy; gianluiginapoli@gmail.com (G.N.); paolo.basile@uniba.it (P.B.); m.c.carella92@gmail.com (M.C.C.); daniele.df93@gmail.com (D.D.F.); micheledavide.latorre@policlinico.ba.it (M.D.L.); marcomatteo.ciccone@uniba.it (M.M.C.); 2 Department of Perioperative Cardiology and Cardiovascular Imaging, Centro Cardiologico Monzino, IRCCS, 20138 Milan, Italy; saima.mushtaq@cardiologicomonzino.it (S.M.); andrea.baggiano@cardiologicomonzino.it (A.B.); gianluca.pontone@cardiologicomonzino.it (G.P.); 3 Department of Biomedical, Surgical and Dental Sciences, University of Milan, 20122 Milan, Italy

**Keywords:** CCTA, coronary plaque, coronary artery calcium

## Abstract

Historically, cardiovascular prevention has been predominantly focused on stress-induced ischemia, but recent trials have challenged this paradigm, highlighting the emerging role of vulnerable, non-flow-limiting coronary plaques, leading to a shift towards integrating plaque morphology with functional data into risk prediction models. Coronary computed tomography angiography (CCTA) represents a high-resolution, low-risk, and largely available non-invasive modality for the precise delineation of plaque composition, morphology, and inflammatory activity, further enhancing our ability to stratify high-risk plaque and predict adverse cardiovascular outcomes. Coronary artery calcium (CAC) scoring, derived from CCTA, has emerged as a promising tool for predicting future cardiovascular events in asymptomatic individuals, demonstrating incremental prognostic value beyond traditional cardiovascular risk factors in terms of myocardial infarction, stroke, and all-cause mortality. Additionally, CCTA-derived information on adverse plaque characteristics, geometric characteristics, and hemodynamic forces provides valuable insights into plaque vulnerability and seems promising in guiding revascularization strategies. Additionally, non-invasive assessments of epicardial and pericoronary adipose tissue (PCAT) further refine risk stratification, adding prognostic significance to coronary artery disease (CAD), correlating with plaque development, vulnerability, and rupture. Moreover, CT imaging not only aids in risk stratification but is now emerging as a screening tool able to monitor CAD progression and treatment efficacy over time. Thus, the integration of CAC scoring and PCAT evaluation into risk stratification algorithms, as well as the identification of high-risk plaque morphology and adverse geometric and hemodynamic characteristics, holds promising results for guiding personalized preventive interventions, helping physicians in identifying high-risk individuals earlier, tailoring lifestyle and pharmacological interventions, and improving clinical outcomes in their patients.

## 1. Introduction

Coronary artery disease (CAD) represents one of the leading causes of mortality and morbidity worldwide, despite significant advances in diagnosis and treatment during the last decades [1,2]. At one end of the spectrum of CAD, there are individuals who have experienced multiple acute coronary events. Conversely, at the opposite end of the spectrum, there are patients with enduring stable angina who have consistently maintained clinical stability. Historically, cardiovascular prevention has been predominantly focused on identifying coronary plaques responsible for stress-induced ischemia, thereby guiding clinical decision making and therapeutic interventions [3]. This approach has been evidenced by studies [4,5,6]. Nevertheless, recent randomized clinical trials (RCTs) have called into question this long-held approach, suggesting a need for a paradigm shift in our clinical practice [7,8]. The PROSPECT study and subsequent investigations have significantly advanced our understanding of high-risk plaques. They have demonstrated that the majority of acute coronary syndromes (ACSs) originate from plaques that were previously considered “stable” based on stress tests [9,10]. A more recent study, the PREVENT trial, demonstrated that preventive PCI of high-risk, non-flow-limiting coronary plaques reduces major adverse cardiac events (MACEs) compared with optimal medical therapy (OMT) alone [7]. Furthermore, emerging evidence indicates that vulnerable plaques may exert systemic effects beyond the local coronary environment, contributing to systemic inflammation, microvascular dysfunction, and the development of a prothrombotic state, which predisposes to adverse cardiovascular events [11]. Consequently, interventions designed to address vulnerable plaque have the potential to not only prevent ACS but also to mitigate the global cardiovascular risk [11].

**Figure 1:**
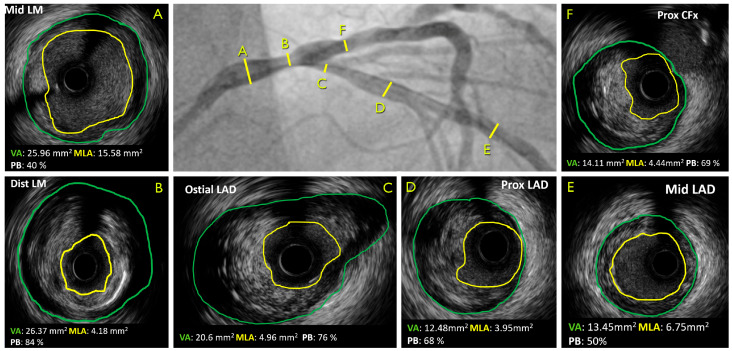
Correspondent ICA and IVUS of a patient with LM distal lesion. CFx, circumflex artery; LAD, left anterior descending artery; LM, left main; MLA, minimum lumen area; VA, vessel area.

The application of advanced imaging techniques, including intravascular ultrasound (IVUS), optical coherence tomography (OCT), and near-infrared spectroscopy (NIRS), has facilitated a profound comprehension of plaque morphology and composition (Figure 1). This has led to the more precise identification of high-risk lesions, thereby enhancing the accuracy of diagnostic procedures [7]. Nevertheless, recent advances in coronary computed tomography angiography (CCTA) have enhanced our capacity to identify and assess plaque vulnerability non-invasively [12,13,14,15,16] (Table 1). CCTA offers high-resolution visualization of the coronary arteries, allowing for the precise delineation of plaque composition, morphology, and inflammatory activity [17,18,19,20,21,22,23,24]. Furthermore, the advent of novel CT-based techniques, such as coronary artery calcium (CAC) scoring and CT-derived fractional flow reserve (FFRCT), has significantly augmented our capacity to stratify high-risk plaque and predict adverse cardiovascular outcomes [22,23,24,25]. The objective of this review is to elucidate the growing role of CCTA in the detection and characterization of vulnerable plaques, with a particular focus on its potential to reshape risk assessment paradigms and inform clinical decision making in the management of CAD.

## 2. Calcium Score

The increasing prevalence of cardiovascular disease (CVD) demands the development of novel methodologies for risk assessment and primary prevention strategies. Among these, coronary artery calcium (CAC) scoring has emerged as a promising tool for predicting future cardiovascular events in asymptomatic individuals [26]. Coronary artery calcification represents a late-stage manifestation of atherosclerosis, wherein hydroxyapatite crystals accumulate within the intima and media layers of the coronary arteries [27]. This calcification deposition is indicative of the chronic inflammatory milieu and endothelial dysfunction that are characteristic of atherosclerotic plaque development. CAC scoring, which is typically performed using non-contrast cardiac CT imaging, allows for the quantification of the extent and density of coronary artery calcification within the coronary wall. A variety of scoring systems, including the Agatston score, volume scores, and mass scores, facilitate the standardized assessment of CAC burden based on lesion size and density (Table 2, graphical abstract) [28]. It is of significant importance to note that CAC scoring demonstrated incremental prognostic value beyond traditional cardiovascular risk factors in terms of myocardial infarction, stroke, and all-cause mortality [26]. Consequently, the use of CAC scoring has become a valuable tool for refining risk stratification in asymptomatic individuals and guiding the implementation of personalized preventive interventions.

Although CAC = 0 is often associated with a low risk of obstructive CAD, certain populations still exhibit non-negligible rates of obstructive CAD despite a zero score, since significant non-calcified plaque requiring treatment to stabilize, delay, or reverse disease progression may be present. Notably, spotty calcifications might be occasionally undetected by the 3 mm slices that are routinely used for CAC assessment by CCTA. This limitation is more pronounced in those under 40, who tend to have a higher burden of non-calcified plaque, making CAC = 0 an unreliable indicator for excluding obstructive CAD in this age group [29].

Given its prognostic utility, there is a growing interest in integrating CAC scoring into existing risk prediction models, such as the Framingham Risk Score (FRS) or the European Society of Cardiology Systemic Coronary Risk Estimation (SCORE) [30]. A risk assessment tool, integrating the CAC with conventional cardiovascular risk factors (CRFs), has been developed and validated externally by McClelland et al. [31]. The integration of CAC into this risk model prediction, tailored for estimating the 10-year risk of coronary heart disease (CHD), resulted in notable enhancements in risk prediction accuracy, as evidenced by the high discrimination and calibration demonstrated in the external validation cohort.

It is noteworthy that the variability in interscan measurements of CAC scoring is estimated to be approximately 10%, which facilitates the quantitative evaluation of CAC progression. In the MESA study, which enrolled 5756 participants and featured an average interval of 2.4 years between CT scans, there was an annual increase in CAC scores of between 20% and 25% [33]. The baseline CAC level is found to exhibit a robust association with subsequent CAC progression, contributing to the skewed distribution of CAC observed. This phenomenon is corroborated by findings from the HNR study [34]. It is notable that the progression of coronary artery calcification (CAC) has been identified as a predictor of heightened risks for myocardial infarction and all-cause mortality. Nevertheless, individuals exhibiting a “double zero” CAC scan, indicative of a CAC score of 0 at both baseline and the subsequent 5-year interval, exhibited the lowest estimated 10-year risk of coronary disease. This observation suggests the potential utility of undertaking a repeat scan after five years, particularly for individuals initially classified as high-risk on the basis of a baseline CAC score equal to or exceeding 400 [35].

Furthermore, the predictive efficacy of CAC scoring was evaluated within a cardiovascular event forecasting model developed through the application of a machine learning algorithm to the MESA dataset [32]. It is noteworthy that the CAC score emerged as the most influential predictor of cardiovascular disease, surpassing over 700 other baseline variables in predictive significance.

Notably, most evidence supporting the use of CAC in risk prediction stems from studies with low rates of statin use, which have informed guidelines for personalized statin therapy. Statin therapy modestly accelerates the calcification of plaques, leading to more stable and lower-risk compositions, and can sometimes increase the Agatston CAC score by raising plaque density. A systematic review and meta-analysis found that while statins do not reduce CAC, they can slow its progression, particularly in individuals with high baseline CAC scores (>400), being more effective in stabilizing advanced coronary lesions. However, the relationship between CAC progression under statin therapy and cardiovascular outcomes is complex, suggesting a need for more intensive primary prevention in individuals with calcification progression [36].

The integration of CAC scoring into primary prevention strategies has the potential to significantly reduce the burden of CVD and healthcare costs associated with unnecessary interventions. Furthermore, it could identify asymptomatic individuals with subclinical atherosclerosis and elevated cardiovascular risk, who could benefit from intensified lifestyle modifications, pharmacological therapies, or referral for further diagnostic evaluation [36,37,38]. To date, there are still challenges to be overcome in terms of defining optimal CAC thresholds for risk stratification and establishing cost-effective strategies for the widespread implementation of CAC scoring in clinical practice. In a subanalysis of the JUPITER trial, patients with a CAC score of 0 had a 5-year rosuvastatin number needed to treat (NNT) of 124, while those with a CAC score greater than 100 had an NNT of 19 [39]. Nasir et al. conducted a study to assess the impact of coronary artery calcium (CAC) on statin allocation in accordance with the 2013 ACC/AHA Cholesterol Treatment Guidelines. Their findings indicated that individuals with a CAC score of 0 exhibited event rates below the treatment thresholds. Conversely, a CAC score of >0 indicated a potential benefit from statin therapy [40]. Furthermore, the use of CAC scoring facilitates the recommendation of prophylactic daily aspirin, with the potential for net harm when CAC = 0 and net benefit when CAC > 100 [41]. Finally, McEvoy et al. demonstrated that there were significant variations in the 10-year number needed to treat (NNT10) for blood pressure targets based on the baseline coronary artery calcium (CAC) status [42]. Consequently, the integration of CAC scoring into risk stratification algorithms has the potential to transform the field of cardiovascular medicine by enabling the development of more effective and targeted preventive interventions and optimizing clinical outcomes. This represents a paradigm shift in the assessment of cardiovascular risk and the delivery of primary prevention strategies. Furthermore, the implementation of CAC scoring could facilitate engagement with individuals in the management of their cardiovascular health. This would involve the provision of tangible evidence of the underlying disease burden, which could then motivate adherence to preventive measures and pharmacological therapies.

## 3. Plaque Morphology

### 3.1. Adverse Plaque Characteristics

**Figure 2:**
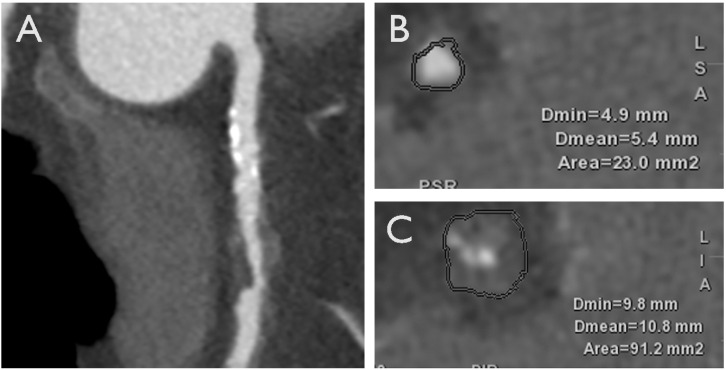
A case of a 70-year-old male with an HRP on proximal right coronary artery with LAP and positive remodeling. HRP, high-risk plaque; LAP, low-attenuation plaque. (**A**): Multiplanar reconstruction (MPR) of the coronary vessel. (**B**,**C**): axial view of the coronary artery at the level of proximal edge of the stenosis and minimal luminal area (MLA) of the stenosis, respectively.

Over the past decade, advances in CCTA have revolutionized the assessment of CAD, enabling detailed characterization of coronary plaque morphology and composition through a progressive higher spatial resolution and improving the identification and characterization of high-risk plaques prone to rupture [43,44,45,46]. The presence of high-risk plaque has been consistently linked to an elevated risk of adverse cardiovascular events, including myocardial infarction and cardiovascular mortality. Furthermore, the extent and severity of coronary plaque burden as assessed by CCTA have been demonstrated to significantly correlate with future cardiovascular outcomes, thereby enhancing risk stratification in patients with CAD [47,48,49]. In individuals presenting with acute chest pain, although flow-limiting stenosis continues to demonstrate significant predictive value for ACS, the presence of high-risk plaque characteristics, including positive remodeling, low attenuation (defined as computed tomography attenuation of <30 Hounsfield units), the napkin-ring sign, and spotty calcification, following adjustment for stenosis ≥50%, has been associated with a substantial nine-fold increase in the likelihood of future ACS [43,50,51]. In the SCOT-HEART trial, a cohort of 1769 participants was monitored over a five-year period. The extent of low-attenuation plaque (LAP) emerged as the most robust predictor of myocardial infarction (MI), with an adjusted hazard ratio (HR) of 1.60 (95% CI: 1.10–2.34 per doubling; *p* = 0.014) (Figure 2, graphical abstract) [52]. This association was observed to be consistent regardless of the individual’s cardiovascular risk score, CAC score, or the presence of flow-limiting stenosis. Consequently, integrating CCTA-derived information about adverse plaque characteristics in the context of their additional prognostic implications could play a pivotal role in guiding revascularization strategies in patients with CAD. This would inform decisions about the necessity for invasive coronary angiography (ICA) and subsequent revascularization procedures [53]. Furthermore, the integration of coronary CT imaging findings into clinical decision-making algorithms enables the implementation of more personalized and tailored approaches to revascularization, thereby optimizing patient outcomes while minimizing procedural risks [54]. Continued research efforts aimed at refining plaque characterization techniques and elucidating the therapeutic implications of coronary CT-derived information are essential for advancing the field of cardiovascular imaging and optimizing patient care.

### 3.2. Adverse Geometric Characteristics

The susceptibility to plaque disruption encompasses a multifaceted interplay between inherent plaque fragility and external hemodynamic influences acting upon the plaque interface. This intricate relationship serves to illustrate the significance of distinct plaque localization and vascular geometrical characteristics in modulating coronary flow dynamics and endothelial shear stress [54,55,56]. Furthermore, the capacity of CCTA to precisely delineate plaque spatial distribution and vessel curvature offers a comprehensive insight into the intricate vascular pathophysiology.

In a substudy of ICONIC (Incident Coronary Syndromes Identified by Computed Tomography), the presence of adverse geometric characteristics, defined as distance from the coronary ostium to lesion, location at vessel bifurcations, and vessel tortuosity (presence of one bend of greater than 90° or three curves of 45° to 90° using a three-point angle within the lesion), were found to be independently associated with a greater risk of future culprit lesion. The hazard ratio for one adverse geometric characteristic was 2.90 (95% confidence interval, 1.38–6.08), and the hazard ratio for two or more adverse geometric characteristics was 6.84 (95% confidence interval, 3.33–14.04). The 90% confidence interval (CI) for the hazard ratio (HR) for ≥2 AGCs was 1.38 to 6.08, with an HR of 6.84 (95% CI, 3.33–14.04) for ≥2 AGCs. Furthermore, the inclusion of AGCs in a predictive model, which already comprised stenosis severity, adverse morphological plaque features, and quantitative plaque metrics, demonstrated additional discriminative efficacy in identifying precursor lesions predisposing to adverse outcomes [57] (graphical abstract).

### 3.3. Adverse Hemodynamic Characteristics

Among coronary plaques exhibiting similar vulnerable characteristics, variations in hemodynamic forces can influence the risk of rupture, as a consequence of an imbalance between plaque’s mechanical strength and internal plaque stress. 

Previous autopsy investigations have demonstrated that increased plaque structural stress (PSS) occurs subsequent to plaque rupture. This is defined as the maximum principal stress normalized by coronary pressure. Empirical evidence indicates that the localization of peak PSS holds predictive value for identifying the site of plaque rupture [58,59]. Furthermore, the composition of the plaque itself exerts an influence on PSS dynamics, as evidenced by studies demonstrating that extensive lipid cores, thin fibrous caps, and superficial microcalcifications collectively contribute to an elevation in PSS levels [60,61]. In the VIVA study, PSS derived from virtual histology IVUS demonstrated a statistically significant elevation in lesions associated with MACE, particularly in high-risk regions (lesions with plaque burden ≥ 70% and/or thin-cap fibroatheroma) [62]. Furthermore, the incorporation of PSS measurements markedly augmented the predictive capacity of virtual histology IVUS for identifying impending MACE in plaques with a plaque burden of at least 70% and a minimal luminal area (MLA) of 4 mm^2^. Furthermore, plaques responsible for MACE exhibited larger superficial calcium deposits, which contributed to a significant elevation in PSS levels.

**Figure 3:**
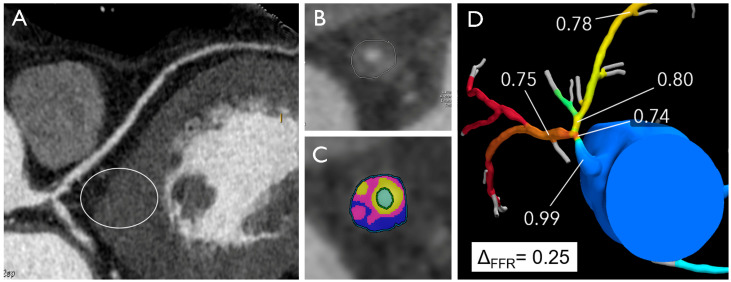
Distal left main artery plaque in a 53-year-old male with atypical chest pain and normal electrocardiogram. The multiplanar reconstruction image (**A**) shows a plaque in distal LM with high-risk features (positive remodeling, low attenuation, high plaque volume) (**B**,**C**) and positive FFRCT with high delta FFR-CT (**D**).

The EMERALD study proposed that integrating four specific non-invasive hemodynamic parameters derived from CCTA could potentially enhance the prediction of ACS risk, thus allowing for the development of optimized treatment strategies for individuals at heightened risk. The following parameters were identified as potential predictors of ACS risk: (1) CTFFR (cut-off value of 0.80), (2) change in CTFFR across the lesion (Δ CTFFR, cut-off value of 0.06), (3) wall shear stress (WSS) (cut-off value of 154.7 dyn/cm^2^), and (4) axial plaque stress (cut-off value of 1606.6 dyn/cm^2^) [63] (Figure 3, graphical abstract).

A total of 72 patients with ACS who underwent CCTA during a two-year period prior to the index event were evaluated using computational fluid dynamics (CFD) to assess the hemodynamic forces acting on 216 coronary lesions (66 culprit and 150 non-culprit) with a diameter stenosis (DS) > 30%.

Consequently, the incorporation of non-invasive hemodynamic assessments into clinical practice has the potential to enhance the identification of culprit lesions that are predisposed to future ACS. By evaluating hemodynamic parameters such as PSS alongside traditional imaging modalities, clinicians can gain deeper insights into the vulnerability and propensity for rupture of atherosclerotic plaques. This comprehensive approach enables a more nuanced risk stratification strategy, which allows for the early detection and targeted management of high-risk lesions.

## 4. Use of CCTA to Facilitate PCI

The spread of CCTA has led to the emerging role of CT in planning percutaneous coronary interventions (PCIs) by providing detailed assessments of atherosclerotic plaques, including their extent, volume, and composition. Unlike invasive coronary angiography (ICA), which primarily evaluates luminal narrowing, CCTA provides a comprehensive overview of plaque morphology and significance without the need for intracoronary imaging modalities (IVUS or OCT), which are used in less than 10% of PCI procedures due to their duration, costs, and the expertise required. As a first-line non-invasive tool in the evaluation of suspected CAD, the integration of CCTA-derived information into the preoperative planning of coronary revascularization presents multiple advantages. Firslty, CCTA precisely characterizes plaque position, length, and calcium content, assessing the functional significance of each stenosis; secondly, it supports the selection of guiding catheters in order to engage coronary arteries by evaluating the position of the ostia and their involvment in CAD; moreover, it is able to estimate myocardial mass at risk of coronary occlusion, assessing the importance of each side branch involved in CAD; neverthless, it could help interventional cardiologists in sizing the stents and also in deciding to apply a plaque-modifying technique because of excessive burden of calcium or in case of extreme tight lesion. Finally, the role of CCTA in guiding revascularization of chronic total occlusion (CTO) has been recently highlighted in an expert consensus document of the Society of Cardiovascular Computed Tomography [64,65]. By providing detailed anatomical information, including the degree of calcification and length, CCTA facilitates precise procedural planning, which can improve successful rates. The integration of CCTA with fluoroscopy further perfects the procedural guidelines, carrying out the display and navigation in real time during the interventions. In addition, the scores dedicated to the CCTA, such as J-CTO scores and the Rector CT, provide procedural results based on CTO morphology and help with the selection of patients and the procedural optimization strategy [66]. Despite some well-known challenges (such as the limitations of spatial resolution for collateral evaluation), constant progress in CCTA technology continues to improve the procedural results and the management of long -term patients, underscoring its pivotal role in modern angioplasty and CTO-revascularization strategies.

## 5. Epicardial and Pericoronary Adipose Tissue

In addition to their role as an energy reserve system, epicardial adipose tissue (EAT) and pericoronary adipose tissue (PCAT) have been shown to significantly contribute to the development of coronary artery calcification, inflammation, and plaque vulnerability [67,68]. The potential of PCAT-CT attenuation, which assesses adipocyte differentiation and size, as well as leukocyte infiltration, to serve as a marker of pericoronary inflammation and to predict the progression of CAD has been demonstrated. This is evidenced by the correlation between PCAT attenuation and plaque development, vulnerability, and rupture. Furthermore, the perivascular fat attenuation index (FAI), a biomarker derived from CCTA, has been shown to be directly proportional to CAD progression, particularly to dynamic changes observed around culprit lesions in ACS [68]. Furthermore, the CRISP-CT study demonstrated that perivascular FAI quantification has an independent and incremental prognostic value, identifying individuals at heightened risk for all-cause and cardiac mortality [69]. The identified perivascular FAI cutoff of ≥−70 HU emerged as a robust indicator, facilitating risk stratification with a five- to nine-fold increased adjusted risk for cardiac death. It is of note that perivascular FAI augmentation significantly enhanced mortality risk discrimination and reclassification beyond existing prognostic models, thereby underscoring its potential as a tool for guiding targeted primary and intensive secondary prevention strategies (graphical abstract).

The integration of PCAT and EAT analysis into cardiovascular risk stratification models has the potential to enhance the predictive capacity for MACEs, thereby revolutionizing primary and secondary prevention strategies. These insights, which have been corroborated by biopsy and in vivo data, herald an intriguing frontier in cardiovascular risk assessment.

## 6. New Perspectives

In the PROMISE trial, the majority of cardiac events occurred in patients with non-obstructive disease [70]. In a more recent study, the PREVENT trial demonstrated that percutaneous coronary intervention (PCI) targeting focal non-flow-limiting (FFR-negative) plaques exhibiting features of vulnerability on intravascular imaging was superior to OMT over a two-year period among patients with stable CAD in terms of death from cardiac causes, target vessel myocardial infarction, ischemia-driven target vessel revascularization, or hospitalization for unstable or progressive angina [7].

Historically, physiological indices such as FFR have been of pivotal importance in guiding decisions regarding revascularization. Nevertheless, these findings prompt questions regarding the relative importance of anatomical versus physiological criteria in the management of CAD. 

In the SCOT-HEART trial, the incorporation of CCTA alongside conventional care among patients experiencing stable chest pain yielded a significant reduction in the incidence of mortality from coronary heart disease or nonfatal myocardial infarction over a five-year period compared to standard care alone [71]. It is noteworthy that this benefit was achieved without a concomitant increase in the rate of ICA or coronary artery revascularization procedures. 

Consequently, the provision of detailed anatomical information and the characterization of plaque morphology by CT facilitates risk stratification and enables the identification of individuals at heightened risk of cardiovascular events. In addition to risk stratification, CT imaging also facilitates the monitoring of disease progression and treatment efficacy over time. Serial CT assessments permit the tracking of changes in plaque burden and composition, thereby facilitating the implementation of personalized adjustments to preventive strategies in accordance with evolving risk profiles [72]. 

Furthermore, CCTA proved highly valuable in the emergency setting for patients with acute chest pain due to its high diagnostic accuracy in detecting CAD. It is effectively able to stratifies patients into different risk categories, which helps in tailoring appropriate treatment strategies and reducing the need for invasive procedures by accurately identifying those with low- to intermediate-risk of CAD, thereby minimizing unnecessary interventions. Its rapid imaging capability supports swift decision making crucial in emergencies and, when combined with high-sensitivity troponin testing, enhances the diagnosis of ACS, especially in ambiguous cases. Additionally, it provides detailed plaque morphology, identifying high-risk plaques that other imaging modalities might miss. Recently, an expert consensus document of the Society of Cardiovascular Computed Tomography, endorsed by the American College of Radiology and North American Society for Cardiovascular Imaging, recommended CCTA as a first-line triage strategy for acute chest pain in patients presenting in the emergency department without a diagnosis of myocardial infarction [73]. Each piece of research also indicates that using CCTA or CMR as an initial step before invasive coronary angiography in cases of non-ST elevation myocardial infarction (NSTEMI) is safe, with comparable rates of hospitalization, major adverse cardiac events, and complications to those seen with routine care [74].

In conclusion, CT imaging represents a valuable tool in directing primary prevention efforts by providing a comprehensive assessment of coronary plaque burden, morphology, and vulnerability. This enables the implementation of tailored preventive interventions aimed at reducing the burden of cardiovascular disease.

## 7. Conclusions

In light of recent advances, CCTA represents a transformative milestone in cardiovascular medicine, offering a paradigm-shifting approach to risk assessment and primary prevention in patients with CAD. The utilization of non-invasive imaging technology enables the generation of valuable insights into the pathophysiology of atherosclerosis and serves as a robust predictor of future cardiovascular events, even in asymptomatic individuals. The integration of CAC scoring, as well as the identification of high-risk plaque morphology, adverse geometric and hemodynamic characteristics, and PCAT evaluation into risk stratification algorithms has yielded promising results in guiding personalized preventive interventions and optimizing clinical outcomes. This paves the way for a new era of precision medicine in preventive cardiology. Furthermore, CT imaging not only assists in risk stratification but also enables the monitoring of disease progression and treatment efficacy over time. Serial CT assessments have been demonstrated to allow for a personalized adjustment of preventive strategies based on evolving risk profiles, thus facilitating tailored preventive interventions aimed at reducing the burden of cardiovascular disease. Finally, recent randomized controlled trials (RCTs), such as the PROMISE and the PREVENT, have challenged the historical reliance on physiological indices alone in guiding revascularization, suggesting a shift towards targeted interventions for non-obstructive but high-risk coronary plaques. Moving beyond the sole focus on stress-induced ischemia, a comprehensive understanding of plaque vulnerability should inform risk stratification algorithms, therapeutic decision making, and personalized treatment strategies.

## Figures and Tables

**Table 1:** Invasive imaging modalities compared to CCTA for detecting vulnerable plaque features.

	IVUS	OCT	NIRS	CCTA
Spatial resolution	20–100 μm	10–15 μm		0.4 mm
Lipid-rich core	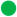 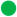 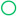	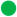 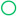 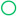	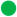 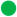 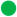	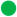 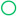 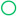
Fibrous cap thickness	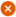	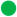 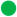 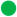	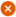	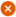
Calcifications	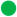 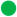 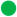	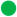 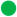 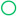	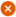	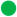 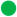 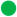 ^(*)^
Positive vessel remodeling	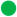 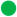 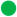	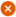	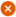	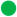 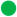 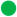
Coronary Inflammation	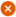	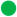 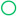 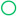	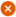	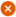
Neovascularization	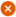	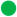 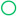 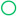	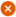	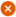
PCAT inflammation	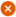	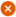	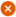	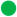 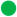 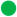

CCTA, coronary computed tomography angiography; IVUS, intravascular ultrasound; NIRS, near-infrared spectroscopy; OCT, optical coherence tomography; PCAT, pericoronary adipose tissue. (*) Not able to distinguish microcalcifications (<1 mm). 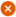 N/A; 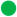

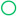

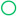
 low; 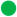

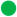

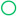
 medium; 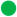

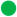

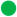
 high.

**Table 2:** Examples of CAC scoring system.

	AGATSON Score	Visual Assessment Score	CAC-DRS
*Calculation*	Sum of the attenuation (in HU) and area of all CAC lesions in the coronary arteries.	Calcium quantitative assessment for each of the main epicardial coronary arteries.	Sum of the Agatston score or visual assessment score for each coronary artery.
*Levels*	0: low risk. 1–99: mildly increased risk. 100–299: moderately increased risk. >300: moderate to severely increased risk.	0: no CAC. 1: mild calcification in less than a third of the coronary artery. 2: moderate calcification involving one-third to two-thirds of the artery. 3: severe calcification involving two-thirds of the artery.	0–3 levels as calculated from AGATSON score (A) or visual assessment (V) score plus the number of affected coronary arteries.
*Interpretation*	Absolute score (in Agatston units). Age-, sex-, and race-specific percentile (derived from the MESA risk score calculator).	Sum of the score for each of the coronary arteries and can be categorized into three categories of severity: 0, 1–3, and 4–12.	An A0-1-2-3/N0-1-2-3-4 score can be derived from Agatston score. An V0-1-2-3/N0-1-2-3-4 score can be calculated from visual assessment score.

CAC, coronary artery calcium; CAC-DRS, CAC Data and Reporting System; HU, Hounsfield unit.

## Data Availability

No new data were created for this review.
